# *GSTP1* and *GSTO1* single nucleotide polymorphisms and the response of bladder cancer patients to intravesical chemotherapy

**DOI:** 10.1038/srep14000

**Published:** 2015-09-10

**Authors:** Xiaheng Deng, Xiao Yang, Yidong Cheng, Xuzhong Liu, Xiao Li, Ruizhe Zhao, Chao Qin, Qiang Lu, Changjun Yin

**Affiliations:** 1 Department of Urology, The First Affiliated Hospital of Nanjing Medical University, 300 Guangzhou Road, Nanjing 210029, P.R. China

## Abstract

SNPs may restrict cell detoxification activity and be a potential risk factor for cancer chemosensitivity. We evaluated the predictive value of these polymorphisms on the sensitivity of bladder cancer patients to epirubicin and mitomycin chemotherapy instillation as well as their toxicities. SNPs were analyzed by TaqMan genotyping assays in 130 patients treated with epirubicin and 114 patients treated with mitomycin. Recurrence-free survival (RFS) was estimated by the Kaplan-Meier method, and hazard ratios (HRs) and 95% confidence intervals (CIs) of the HRs were derived from multivariate Cox proportional hazard models. *GSTP1* rs1695 and *GSTO1* rs4925 were also associated with RFS in the epirubicin group. Patients carrying the *GSTP1* AG+GG and *GSTO1* AC+AA genotypes had an unfavorable RFS. Patients with the *GSTP1* AA and *GSTO1* CC genotypes had a reduced risk of recurrence after the instillation of epirubicin. In addition, patients with the *GSTP1* rs1695 AA genotype had an increased risk of irritative voiding symptoms; while patients with the *GSTO1* rs4925 CC genotype had a decreased risk of hematuria. Our results suggest that *GSTP1* and *GSTO1* polymorphisms are associated with epirubicin treatment outcomes as well as with epirubicin-related toxicity.

Bladder cancer is the most common malignancy of the urinary tract, with 74,690 cases and 15,580 deaths in the United States in 2013^1^. Notwithstanding multidisciplinary advances in its treatment, bladder cancer continues to have an unacceptably high morbidity and mortality^2^. Approximately 80% of all patients with bladder cancer initially present with superficial tumors, of which 12.5% progress to invasive disease^3^,^4^. Symptoms of early bladder cancer that alert patients to seek medical advice include hematuria and urinary frequency. For patients suspected of having bladder cancer, cystoscopy and transurethral resection are used for diagnosis as well as for total endoscopic tumor resection. However, for patients with grade Ta or T1 lesions, tumor recurrence may present a major problem. Twenty percent of patients with low-risk disease and 40% with medium-risk disease will develop tumor recurrence within one year after transurethral resection of the bladder tumor. Nevertheless, patients with high-risk disease will present a greater recurrence rate (90%) at 2 years after transurethral resection^5^. Intravesical therapy is the most commonly used therapeutic approach for bladder cancer, whereby chemical agents are instilled into the bladder to improve local control and decrease the risk of cancer progression.

Various chemotherapeutics have been administered intravesically to manage superficial bladder cancer. It has been shown that intravesical chemotherapy can effectively reduce disease recurrence within the first 1–5 years after tumor resection^6^. Chemotherapeutic agents, such as thio-tepa, doxorubicin, epirubicin, mitomycin (MMC), or Bacillus Calmette-Guerin (BCG), often have been utilized as prophylactic treatment to prevent tumor recurrence^7^,^8^,^9^. Although the intravesical instillation of BCG has been shown to be more effective than chemotherapeutic agents for prophylactic treatment^7^, BCG has the disadvantage of causing various and frequent local or systemic side effects. Besides, BCG is not an easily accessible instillation agent in China. Epirubicin, an anthracycline-containing drug and a stereoisomer of doxorubicin, has been considered the standard treatment for various cancers. Moreover, it has been shown that epirubicin has slightly fewer adverse drug reactions than other agents^10^. Indeed, Kurth *et al.*^11^ have shown a direct relationship between the epirubicin dose and its effect on tumors *in situ* as well as side effects caused by treatment. Thus, epirubicin is also very frequently used to treat bladder cancer patients due to its few and mild complications. Another standard chemotherapeutic agent, MMC, is the most frequently used chemotherapeutic agent to treat superficial bladder cancer because of its low therapeutic concentration recommended for intravesical infusion^8^.

The clinical response to chemotherapy or instillation agents is influenced by both genetic and environmental factors. Interindividual differences in pharmacokinetics and pharmacodynamics play a primordial role in the response and toxicity profile of different agents. Furthermore, drug absorption, distribution, metabolism, and excretion are controlled by various genetic factors. Glutathione S-transferases (GSTs) are a class of detoxification enzymes that catalyze the conjugation of potentially damaging chemical mutagens to glutathione and protect against the products of oxidative stress; therefore, they are considered as the most important phase II metabolizing enzymes^12^. Studies have shown that upregulated GST activity is a hallmark of a malignant bladder cancer phenotype and is involved in the maintenance of the prooxidant–antioxidant balance towards a more reduced state during tumor progression^13^. GSTP1 has a high level of expression in the bladder and plays a central role in the inactivation of toxic and carcinogenic compounds^14^. *GSTP1* allelic variants may cause an increased susceptibility to oxidative DNA damage and to the accumulation of DNA base adducts^15^. The tendency of GST polymorphisms to alter carcinogen metabolism is well established, with *GSTP1* polymorphisms having been extensively studied in humans. *GSTP1* rs1695, which is characterized by an A-to-G transition at nucleotide 313 (codon 105, exon 5), can cause an isoleucine-to-valine change (Ile105Val)^16^. The *GSTP1* rs1695 polymorphism significantly influences the enzymatic activity and is linked to the clinical outcome of patients who receive platinum-based chemotherapy^17^. The GSTP1 enzyme having Val105 shows a catalytic efficiency for the diol epoxides of polycyclic aromatic hydrocarbons that is seven-fold higher than the isoenzymes having Ile105. Furthermore, the *GSTP1* GG genotype was found to be 2–3 times less stable than the AA genotype and was associated with a higher hydrophilic DNA adduct level^18^.

Both *GSTO1* and *GSTO2* are composed of 6 exons and are separated by 7.5 kb on chromosome 10q24.3. *GSTO2* has 64% aminoacid identity with *GSTO1*^19^. GSTO1 has glutathione-dependent thiol transfer and dehydroascorbate reductase activities^20^ and may play a protective role against calcium-induced apoptosis^21^. GSTO2 also exhibited glutathione-dependent thiol transferase and dehydroascorbate reductase activities. Differently from GSTO1, GSTO2 has a high catalytic activity with 1-chloro-2,4-dinitrobenzene (CDNB) and may play a role in cellular signaling^22^. *GSTO1* rs4925 polymorphism has been found to be associated with age-onset Alzheimer and Parkinson diseases^23^ and a risk of human cancers, such as hepatocellular carcinoma, cholangiocarcinoma, and breast cancer^24^. It has recently been suggested that *GSTO1* and *GSTO2* gene polymorphisms might influence the level of oxidative stress in acute promyelocytic leukemia^25^. However, *GSTO2* rs156697 has been identified as one of the polymorphisms in the *GSTO2* gene. The association of *GSTO2* polymorphism with human cancers, such as hepatocellular carcinoma, colorectal and ovarian cancer has been demonstrated but it appears not to be significantly associated with cancer risk. Moreover, Djukic *et al.* have shown that the *GSTO1* rs4925 and *GSTO2* rs156697 polymorphism is associated with a worse prognosis and a shorter survival in muscle-invasive bladder cancer patients. In the same study, the *GSTP1* Ile allele carriers exhibited an increased overall mortality risk^12^. ATP-binding cassette transporters are transmembrane proteins responsible for most of the drug transport across membranes^26^. It has been shown that polymorphisms in ATP-binding cassette subfamily B (MDR/TAP), member 1 (*ABCB1*) may restrict the cell detoxification activity and be a potential risk factor for cancer chemosensitivity^27^.

More than 90% of patients with a low-grade, noninvasive urothelial tumor (Ta) never progress to having a tumor that invades the basement membrane^28^. Therefore, it is of greater importance to explore the mechanisms of instillation agent toxicity and complications than the risks of metastasis or death from this tumor. Indeed, previous studies have shown the importance of several polymorphisms within candidate genes that are associated with clinical response and toxicity of instillation agents^29^.

In order to confirm and further examine the influence of genetic variations on the clinical outcome and toxicity of intravesical instillation agents, the aim of this study was to examine the *GSTP1*, *GSTO1*, *GSTO2*, and *ABCB1* polymorphisms in a cohort of Chinese bladder cancer patients treated with epirubicin or MMC.

## Methods

In this prospective study, a total of 244 patients diagnosed with nonmuscle invasive bladder cancer were recruited from the First Affiliated Hospital of Nanjing Medical University (Nanjing, China) from May 2007 to October 2012. All patients or patients’ representatives, if direct consent could not be obtained, provided written informed consent to participate in this study, and the study was approved by the Institutional Review Board of Nanjing Medical University (Nanjing, China). All experiments were performed in accordance with relevant guidelines and regulations, and the Institutional Review Board of Nanjing Medical University had approved all experiments. Among them, 130 patients were treated with epirubicin and the other 114 patients were instilled with MMC to prevent cancer recurrence. Prior to recruitment, all subjects were personally interviewed to collect demographic data and clinical characteristics, including age, gender, tobacco use, alcohol use, and self-reported family history of cancer. Patients were excluded from the study if they had a previous history of cancer, had metastasized cancer from other or unknown origins, or were previously subjected to radiotherapy or chemotherapy.

The tumor histological grade was assessed according to the recently published World Health Organization (WHO) consensus^30^. The pathology slides from radical nephrectomy or core biopsy were independently reviewed by two pathologists and were identified as bladder cancer. According to the histopathological grade (WHO 2004, grading of urothelial papilloma)^31^, the patients were classified into two subgroups: low risk and high risk. Individuals who smoked daily for more than 1 year were defined as smokers, and the rest were considered as nonsmokers. Individuals who drank alcohol at least three times per week for more than 6 months were defined as drinkers, and the rest were considered as nondrinkers.

Epirubicin or MMC was instilled into the bladder within 24 h after transurethral resection of the bladder tumor. Thereafter, patients were treated with epirubicin (50 mg/week) or mitomycin (30 mg/week) for 8 weeks, and this dosage was maintained for 12 or more months. The survival time was calculated from the date of confirmed diagnosis until the date of the last follow-up or recurrence. The date of recurrence was obtained from inpatient and outpatient records or from the patients’ families via follow-up telephone calls. The patients who did not suffer from recurrence by the last follow-up date were considered as having nonrecurrent disease.

### Single nucleotide polymorphism (SNP) selection and genotyping

The SNPs included in this study were selected based on previous studies that demonstrated their association with pharmacosensitivity (*GSTP1* rs1695, *GSTO1* rs4925, *GSTO2* rs156697, *ABCB1* rs3747802, and *ABCB1* rs3213619). Genomic DNA of each individual was extracted from 150 μL of EDTA-anticoagulated peripheral blood samples by using a DNA extraction kit (Tiangen Biotech, Beijing, China), according to the manufacturer’s instructions. The polymorphisms were genotyped using a TaqMan SNP genotyping assay (Applied Biosystems, Foster City, CA, USA) and a 384-well ABI 7900HT real-time PCR system (Applied Biosystems, Foster City, CA, USA). A 1-μg sample of total DNA was used for genotyping with primers (Invitrogen, Karlsrule, Germany) (shown in the Supplementary data). SDS 2.4 software (Applied Biosystems, Foster City, CA, USA) was used for allelic discrimination. Each sample was run in triplicate. For quality control, four negative controls were included in each plate, and 5% of the samples were randomly selected for repeated genotyping to verify the results; all of the results were 100% consistent. Primers, probes, and reaction conditions for each SNP analysis are available upon request. Amplification was performed under the following conditions: 50 °C for 2 min; 95 °C for 10 min; and 45 cycles of 95 °C for 15 s and 60 °C for 1 min.

### Statistical analysis

Recurrence-free survival (RFS) was defined as the time from the first instillation of epirubicin or MMC to the first recurrence of bladder cancer. RFS was estimated by the Kaplan–Meier method, and the log-rank test was used to compare different survival curves. Hazard ratios (HRs) and 95% confidence intervals (CIs) of the HRs were derived from univariate and multivariate Cox proportional hazard models. All analyses were carried out using SPSS 13.0 software (IBM, Armonk, NY, USA). A two-sided *P* value < 0.05 represented a statistically significant result.

## Results

### Characteristics of the study population

The frequency distributions of selected characteristics of the cases are shown in Table 1. The median period of instillation treatment was 18 months among the 130 patients who received epirubicin and 17 months among the 114 patients who received MMC regimens. Among all patients, 90 patients had recurrent disease and 5 patients died due to bladder cancer.

### RFS of patients receiving epirubicin or MMC treatment

**Figure 1:**
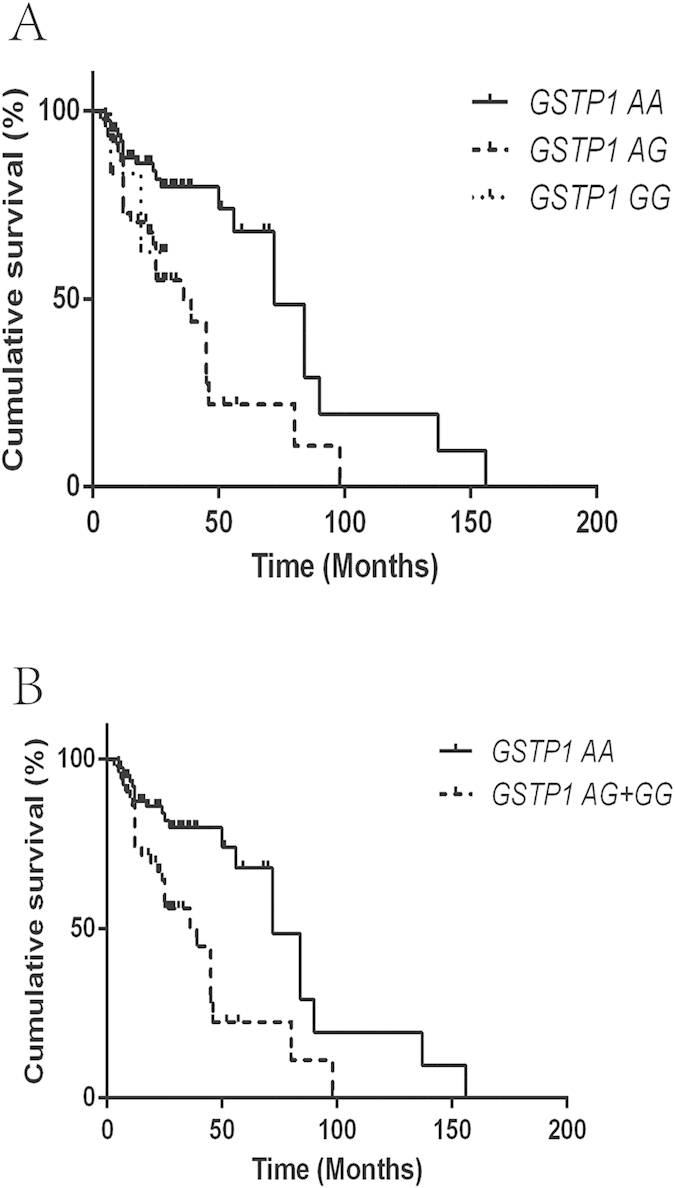
(**A**) The Kaplan–Meier curve representing the association between the *GSTP1* rs1695 genotypes and recurrence-free survival of patients treated with epirubicin. (**B**) The Kaplan–Meier curve representing the association between the *GSTP1* rs1695 AA vs. AG+GG genotype and recurrence-free survival of patients treated with epirubicin.

**Figure 2:**
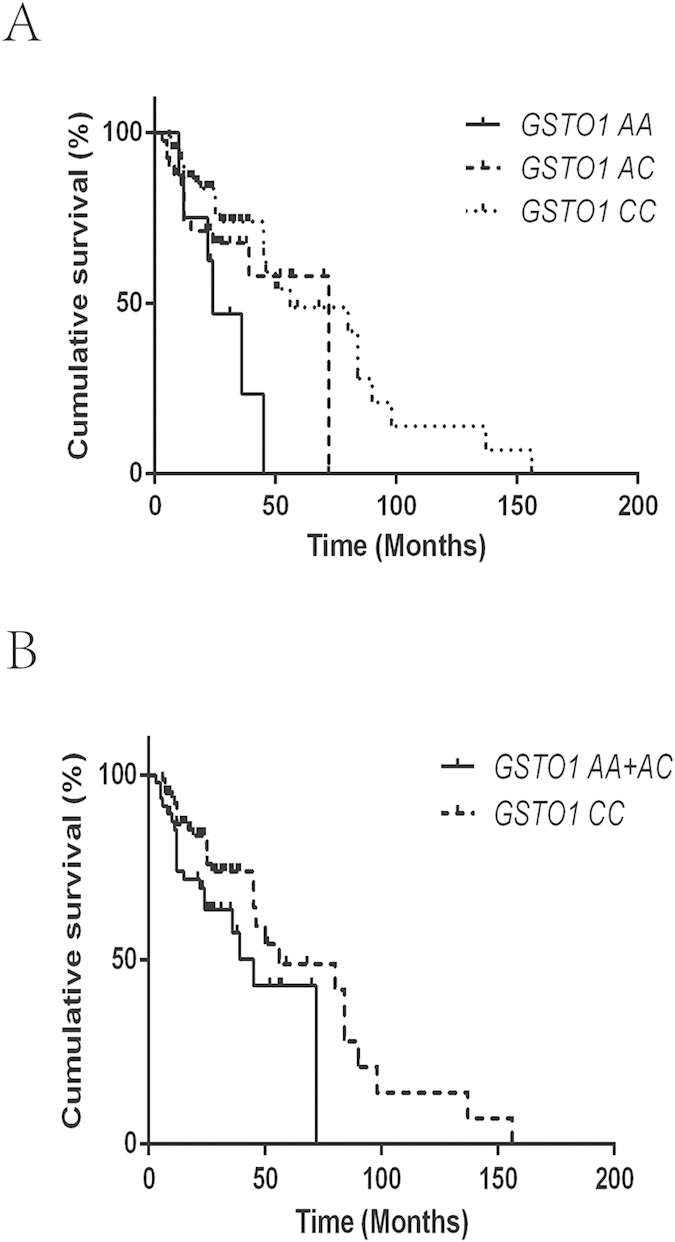
(**A**) The Kaplan–Meier curve representing the association between the *GSTO1* rs4925 genotypes and recurrence-free survival of patients treated with epirubicin. (**B**) The Kaplan–Meier curve representing the association between the *GSTO1* rs4925 AA+AC vs. CC genotype and recurrence-free survival of patients treated with epirubicin.

**Figure 3:**
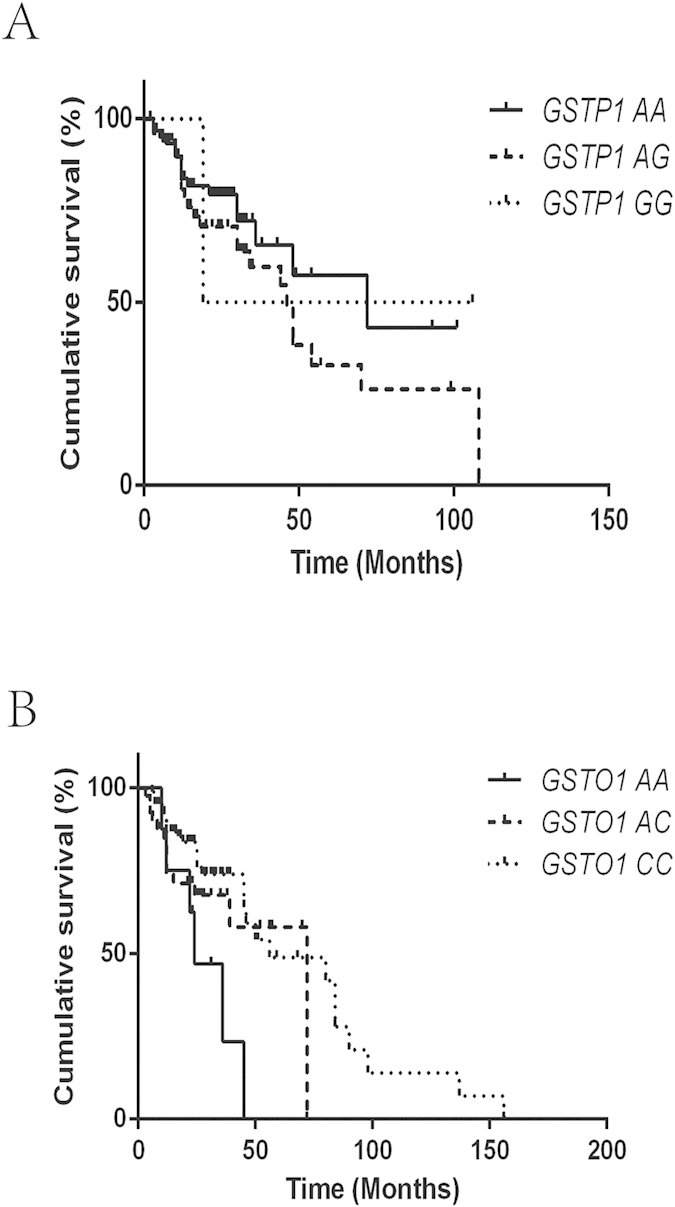
(**A**) The Kaplan–Meier curve representing the association between the *GSTP1* rs1695 genotypes and recurrence-free survival of patients treated with MMC. (**B**) The Kaplan–Meier curve showing the association between the *GSTO1* rs4925 genotypes and recurrence-free survival of patients treated with MMC.

The mean survival time (MST) of patients in the epirubicin-treated group was 29.1 months, while the MST in the MMC-treated group was 27.7 months. The association of RFS with different polymorphisms is presented in Table 2. In our study, the two polymorphisms *GSTP1* rs1695 and *GSTO1* rs4925 were significantly associated with the RFS of patients treated with epirubicin (Table 2). The MST of patients with the *GSTP1* AA genotype was 76.7 months, while the MST of patients with the AG or GG genotype was 40.4 and 23.3 months, respectively (the Log-rank *P* = 0.002) (Fig. 1A). Furthermore, a significant difference in the MST of patients with the AA genotype vs. those with the AG+GG genotype was observed (MST = 40.7 months for the AG+GG genotype, Log-rank *P* = 0.001) (Fig. 1B). Compared to patients with the AA genotype, patients with the AG+GG genotype exhibited a high risk for bladder cancer recurrence (HR = 3.47, 95% CI = 1.75–6.89). Moreover, patients with the *GSTO1* CC genotype were demonstrated to have a longer MST, and this difference was statistically significant (MST = 28.2 months for the AA genotype; 49.0 months for the AC genotype; and 67.4 months for the CC genotype; Log-rank *P* = 0.019) (Fig. 2A–B). Compared to patients with the AC+AA genotype, patients with the CC genotype had a lower risk for bladder cancer recurrence (HR = 0.51, 95% CI = 0.27–0.95). No significant association was found between the *GSTO2* and *ABCB1* polymorphisms and the risk of bladder cancer recurrence with intravesical epirubicin chemotherapy (Table 2). In addition, no association of the examined polymorphisms with the RFS of patients treated with MMC was observed (Fig. 3A–B).

In the epirubicin-treated patients, we also found that *GSTP1* rs4925 in the G-allele carrier patients was significantly associated with a favorable overall survival (MST = 40.4 months for the AA genotype and MST = 36.7 months for the AG+GG genotype; Log-rank *P* = 0.018). However, since the number of deaths was so small, we were not able to draw any convincing conclusions (supplementary Table).

### Combined effect of selected polymorphisms and the RFS of patients receiving epirubicin treatment

**Figure 4:**
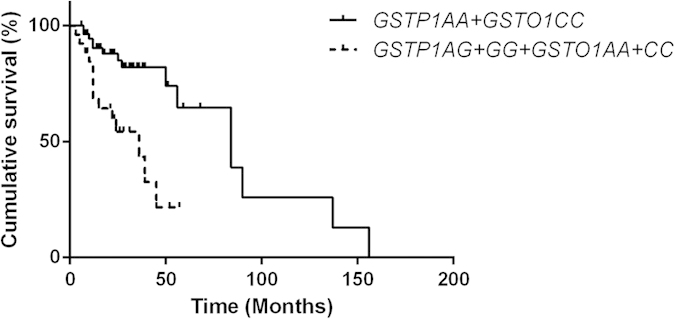
The Kaplan–Meier curve representing the association between the combined *GSTP1* rs1695 and *GSTO1* rs4925 genotypes and recurrence-free survival of patients treated with epirubicin.

In addition to analyzing the influence of separate SNPs, we also examined the effect of combined protective genotypes (the *GSTP1* AA genotype and the *GSTO1* CC genotype) on RFS of patients receiving epirubicin treatment (Table 3). A significantly reduced risk of bladder cancer recurrence was found in patients simultaneously carrying the *GSTP1* AA and *GSTO1* CC genotypes, when compared to patients carrying other combinations of genotypes (Fig. 4) (HR = 0.21, 95% CI = 0.08–0.56).

### Polymorphisms and toxicities of epirubicin and MMC

The occurrence of toxic side effects, including hematuria, irritative voiding symptoms, and suprapubic pain, during treatment with instillation agents is presented in Table 4. Interestingly, a significant association was found between the *GSTP1* rs1695 and *GSTO1* rs4925 genotypes and the prevalence of hematuria and irritative voiding symptoms in the epirubicin-treated group. In our study, the *GSTP1* AA genotype was found to cause a five-times higher risk of irritative voiding symptoms than the AG or GG genotype (OR = 0.20, 95% CI = 0.08–0.49; Table 5). Moreover, the *GSTO1* CC genotype was found to be associated with a reduced risk of hematuria in patients receiving epirubicin (OR = 0.34, 95% CI = 0.12–0.96; Table 5). No association of combined genotypes with toxic side effects was observed for patients receiving MMC.

## Discussion

In the present study, we investigated the possible association between SNPs in the *GSTP1*, *GSTO1*, *GSTO2*, and *ABCB1* genes with response to treatment in patients with bladder cancer. To the best of our knowledge, this is the first study to explore the influence of genetic variants on the clinical outcomes and toxic effects in bladder cancer patients treated with epirubicin or MMC. Overall, we found that patients with the *GSTP1* rs1695 AA genotype had a lower risk of tumor recurrence when treated with epirubicin, but not with MMC ((AG+GG)/AA: HR = 3.47, 95% CI = 1.75–6.89). In addition, patients carrying two C alleles for *GSTO1* rs4925 had a lower risk of bladder cancer recurrence (CC/(AC+AA): HR = 0.51, 95% CI = 0.27–0.95). However, no significant association was found between the *GSTO2* and *ABCB1* polymorphisms and the risk of bladder cancer recurrence in patients receiving intravesical epirubicin chemotherapy.

Since the first evidence that GSTs are involved in the response to chemotherapy^32^, the inconsistent nature of this relationship has been investigated both *in vivo* and *in vitro*^33^,^34^. GSTs catalyze the first step in the formation of mercapturic acids, originating from the elimination pathways of toxic compounds as well as chemotherapy and instillation agents. A study by Stoehlmacher *et al.*^17^ has suggested that GSTP1 may be an important player in the metabolism of platinum drugs in colorectal cancer patients. Furthermore, apoptosis inhibition mediated by the GSTP1–JNK interaction has been found to be the key mechanism in the progression of bladder cancer^35^. Previous studies have reported an effective role of *GSTP1* rs1695 in terms of the clinical outcome of breast cancer patients^36^ and a significant association between high GSTP1 expression of tumor cells and reduced sensitivity to chemotherapy^37^,^38^. The results of our study showed that patients with the *GSTP1* AA genotype had a significantly reduced risk of tumor recurrence after receiving epirubicin. Similarly, others have shown that the *GSTP1* AA genotype also diminishes the risk of chemoresistance to doxorubicin in osteosarcoma patients^39^.

Unlike other GSTs, GSTO1 has a unique structure and function. Rather than having a catalytic serine or tyrosine residue in the active site, GSTO1 utilizes a hyperreactive catalytic cysteine nucleophile^40^. It has been shown that the *GSTO1* CC genotype may modify the risk of developing breast cancer, urothelial carcinoma, and other tumors^41^. In addition, it has been shown that after melanoma cancer cells are treated with a GSTO1 inhibitor, they have a heightened sensitivity to the cytotoxic effects of cisplatin^42^. *GSTO1* rs4925 AC genotype creates a non-conservative amino acid change from hydrophobic to hydrophilic residue, causing the thiol transferase activity of the variant type to reduce to 75% of the wild type, indicating the rs4925 C allele may decrease protection against cellular oxidation stresses caused by instillation agents^43^. Therefore, it may be inferred that the biological effects of the rs4925 CC genotype can be explained by inefficient dehydroascorbate reductase capacity in response to improved chemotherapy sensitivity, which results in induced apoptosis of bladder tumor cells. Our results showed that patients with the *GSTO1* CC genotype had a significantly abridged risk of bladder tumor recurrence (CC/(AC+AA): HR = 0.51, 95% CI = 0.27–0.95) after treatment with epirubicin, which was in agreement with the results of these studies.

In the present study, we also examined the joint effect of multiple genotypes on the risk of bladder cancer recurrence. Based on the genotyping analysis results of selected SNPs in the *GSTP1* and *GSTO1* genes and their association with the treatment outcome, we conducted a combined polymorphism analysis. The results of this combined analysis demonstrated that patients carrying both the *GSTP1* rs1695 AA and *GSTO1* rs4925 CC genotypes had a significantly decreased risk of bladder cancer recurrence, when compared with the combined increased risk genotypes (the *GSTP1* AG+GG genotype and the *GSTO1* AC+AA genotype). This result suggests that crosstalk between these two GST genes might modulate the susceptibility toward bladder cancer recurrence in patients receiving epirubicin. No significant associations of analyzed SNPs were observed in patients treated with MMC. One of the reasons for these findings may be that *GST*s and *ABCB1* do not participate in the metabolism of MMC. It has been clearly demonstrated that *NQO1*, but not the genes we mentioned, plays a pivotal role in MMC activation^44^.

Toxicity is the main dose-limiting factor in cancer therapy. Thus, it is important to assess qualitative and pharmacogenetic associations of the induced side effects when comparing different treatments. Several clinical studies have shown moderate-to-severe acute complications following the instillation of epirubicin or MMC^45^,^46^. In this study, we found that patients with the *GSTP1* rs1695 AA genotype had a 5-fold higher risk of irritative voiding symptoms than patients with the *GSTP1* AG+GG genotype in the epirubicin-treated group. Nevertheless, *GSTP1* rs1695 did not influence the outcome of the MMC-treated group. These findings are in agreement with the results reported in breast cancer patients by Zhang *et al.*^29^. GSTP1 is associated with the regulation of stress signaling and resistance to apoptosis by mechanisms independent of its catalytic activity; and redox-active GSTP1 components inhibit c-JNK, which triggers the apoptotic cascade in the cell. Namely, it has been clearly shown that GSTP1 is involved in the regulation of the JNK signaling pathway in order to protect bladder epidermal cells from epirubicin peroxidation toxicity^47^. Assuming that the *GSTP1* rs1695 AA genotype leads to decreased gene function, it may mimic the inhibition of GSTP1 and confer susceptibility to irritative voiding symptoms. Besides, we also found that patients carrying the *GSTO1* rs4925 AA+AC genotype had an almost 3-fold higher risk of hematuria compared to patients with the CC genotype. Kim *et al.* have demonstrated that GSTO1 might protect against drug toxicity via the regulation of ATP synthase activity^48^. GSTO1 would presumably result in deficient dehydroascorbic acid reductase activity and a lower ascorbic acid level in bladder cancer^49^. As a synergistic nutrient of antineoplastic drugs, ascorbic acid may increase the cytotoxic effects of some anticancer drugs; in particular, anthracyclines are related to free radical formation because of their cytotoxic activities^50^,^51^. Consequently, associated with decreased dehydroascorbate reductase activity, the *GSTO1* rs4925 wildtype CC genotype would reduce lower amount of ascorbic acid than other rs4925 genotypes, which would decrease more free radical production of epirubicin and thus decease the toxicity of instillation drugs. In this study, we did not observe any association of the examined polymorphisms with the antitoxicity of MMC.

Taken together, our results suggest that the *GSTP1* rs1695 and *GSTO1* rs4925 polymorphisms are associated with clinical outcomes of epirubicin treatment but not with MMC treatment. Additionally, the combined effects of *GSTP1* and *GSTO1* polymorphisms showed a significant influence on the RFS in patients receiving epirubicin treatment. Moreover, the *GSTP1* and *GSTO1* polymorphisms were also associated with epirubicin-related toxicities. If our findings are to be confirmed in a larger cohort of patients, the stratification of patients according to the selected SNP genotypes could possibly provide the basis for their individualized treatment.

## Additional Information

**How to cite this article**: Deng, X. *et al.* *GSTP1* and *GSTO1* single nucleotide polymorphisms and the response of bladder cancer patients to intravesical chemotherapy. *Sci. Rep.* **5**, 14000; doi: 10.1038/srep14000 (2015).

## Supplementary Material

Supplementary Information

## Figures and Tables

**Table 1:** Patient characteristics.

		**Patients (n = 244)**
Age (years)		67 ± 12
Gender	Male	189 (77.5%)
	Female	55 (22.5%)
Smoking status	Nonsmoker	190 (77.9%)
	Smoker	54 (22.1%)
Drinking status	No Yes	225 (92.2%) 19 (7.8%)
Family history of cancer	No	239 (98.0%)
	Yes	5 (2.0%)
Tumor number	1	171 (70.1%)
	≥2	73 (29.9%)
Tumor diameter	<3 cm	217 (88.9%)
	≥3 cm	27 (11.1%)
Tumor grade	Low risk	143 (58.6%)
	High risk	101 (41.4%)

**Table 2:** Association between different polymorphisms and recurrence-free survival of patients.

	**Epirubicin**					**MMC**				
	**Recurrence**	**All patients**	**MST** *	**Log-rank** ***P***	**Adjusted HR (95% CI)**	**Recurrence**	**All patients**	**MST** *	**Log-rank** ***P***	**Adjusted HR (95% CI)**
*GSTP1* rs1695										
AA	22	78	76.7	**0.002**	1.00 (reference)	16	61	64.4	0.408	1.00 (reference)
AG	26	46	40.4		**3.29 (1.63–6.63)**	23	51	52.6		0.63 (0.05–7.46)
GG	2	6	23.3		**5.18 (1.05–25.62)**	1	2	62.5		1.56 (0.80–3.07)
AA	22	78	76.7	**0.001**	1.00 (reference)	16	61	64.4	0.218	1.00 (reference)
AG+GG	28	52	40.7		**3.47 (1.75–6.89)**	24	53	53.9		1.54 (0.79–3.01)
*GSTO1* rs4925										
CC	29	82	67.4	**0.019**	1.00 (reference)	26	68	55.1	0.231	1.00 (reference)
AC	15	40	49.0		1.89 (0.94–3.83)	6	30	74.5		0.34 (0.11–1.09)
AA	6	8	28.2		**3.23 (1.16-8.94)**	8	16	45.3		1.12 (0.38–3.33)
CC	29	82	67.4	**0.042**	1.00 (reference)	26	68	55.1	0.404	1.00 (reference)
AC+AA	21	48	43.8		**1.96 (1.05–3.70)**	14	46	62.8		1.20 (0.60–2.44)
*GSTO2* rs156697										
CC	8	15	35.4	0.170	1.00 (reference)	9	21	51.9	0.550	1.00 (reference)
CT	16	50	51.4		0.88 (0.31**–**2.51)	9	38	63.2		0.75 (0.22–2.62)
TT	26	65	67.7		0.51 (0.22–1.22)	22	55	55.3		1.12 (0.48–2.63)
CC	8	15	35.4	0.071	1.00 (reference)	9	21	51.9	0.778	1.00 (reference)
CT+TT	42	115	64.3		0.56 (0.25–1.26)	31	93	59.9		0.94 (0.42–2.14)
*ABCB1* rs3747802										
TT	48	126	28.1	0.580	1.00 (reference)	38	111	28.0	0.105	1.00 (reference)
TC	2	3	71.3		0.33 (0.05–1.99)	2	2	22.0		4.81 (0.99–23.50)
CC	0	1	21.0		0.00	0	1	8.0		0.00
*ABCB1* rs3213619										
TT	47	114	29.1	0.335	1.00 (reference)	36	103	25.8	0.323	1.00 (reference)
TC	3	9	32.7		0.91 (0.27–3.09)	3	6	31.2		0.46 (0.06–3.75)
CC	0	7	23.7		0.00	1	5	64.0		1.17 (0.33–4.17)

^*^Mean survival time was defined as the mean time before recurrence.

**Table 3:** Association between combined *GSTP1* and *GSTO1* polymorphisms and the recurrence-free survival of patients treated with epirubicin.

	**Epirubicin**		
	MST*	**Log-rank** ***P***	**Adjusted HR (95% CI)**
*GSTP1* and *GSTO1*			
AG+GG and AC+AA	31.3		1.00 (reference)
AG+GG and CC	43.3	0.449	0.61 (0.26–1.40)
AA and AC+AA	57.0	0.051	0.30 (0.09–1.08)
AA and CC	83.4	**0.001**	**0.21 (0.08–0.56)**

^*^Mean survival time was defined as the mean time before recurrence.

**Table 4:** The frequencies of hematuria, irritative voiding symptoms, and suprapubic pain due to the toxicity of epirubicin or MMC.

	**Epirubicin**			**MMC**		
	Hematuria (%)*	**Irritative voiding symptoms (%)**	**Suprapubic pain (%)**	**Hematuria (%)**	**Irritative voiding symptoms (%)**	**Suprapubic pain (%)**
GSTP1 rs1695						
AA	12 (9.2)	40 (30.8)	14 (10.8)	11 (9.6)	9 (7.9)	10 (8.8)
AG	6 (4.6)	11 (8.5)	12 (9.2)	10 (8.8)	7 (6.1)	7 (6.1)
GG	1 (0.8)	1 (0.8)	0 (0)	0 (0)	0 (0)	0 (0)
GSTO1 rs4925						
AA	1 (0.8)	2 (1.5)	1 (0.8)	1 (0.9)	2 (1.8)	2 (1.8)
AC	10 (7.7)	16 (12.3)	12 (9.2)	5 (4.4)	2 (1.8)	7 (6.1)
CC	8 (6.2)	34 (26.2)	13 (10)	15 (13.2)	12 (10.5)	8 (7.0)

^*^The number of patients with individual complications versus the number of patients with different instillation agents.

**Table 5:** Association between the *GSTP1* and *GSTO1* polymorphisms and the toxicity of epirubicin or MMC.

	**Hematuria**		**Irritative voiding symptoms**		**Suprapubic pain**	
	**OR (95%CI)**	***P***	**OR (95%CI)**	***P***	**OR (95%CI)**	***P***
Epirubicin						
GSTP1 rs1695 AG+GG/AA	1.08 (0.38–3.07)	0.883	**0.20 (0.08–0.49)**	**<0.001**	1.32 (0.52–3.31)	0.559
GSTO1 rs4925 CC/AA+AC	**0.34 (0.12–0.96)**	**0.042**	1.25 (0.55–2.86)	0.594	0.52 (0.21–1.32)	0.168
MMC						
GSTP1 rs1695 AG+GG/AA	1.13 (0.42–3.00)	0.81	0.81 (0.27–2.43)	0.70	0.75 (0.26–2.18)	0.596
GSTO1 rs4925 CC/AA+AC	2.27 (0.74–6.67)	0.152	2.00 (0.55–7.14)	0.294	0.52 (0.17–1.59)	0.248
