# Supplementary material for: GSTP1 and GSTO1 single nucleotide polymorphisms and the response of bladder cancer patients to intravesical chemotherapy
Source: Sci Rep. 2015 Sep 10;5:14000. doi: 10.1038/srep14000 (PMC4564850; doi:10.1038/srep14000)
Supplement: Supplementary Information [file srep14000-s1.doc]

*GSTP1* and *GSTO1* single nucleotide polymorphisms and the response of bladder cancer patients to intravesical chemotherapy

Authors: Dr. Xiaheng Deng*, Dr. Xiao Yang*, Dr. Yidong Cheng*, Dr. Xuzhong Liu, Dr. Xiao Li, Dr. Ruizhe Zhao, Dr. Chao Qin, Prof. Qiang Lu#, Prof. Changjun Yin

* Xiaheng Deng, Xiao Yang, and Yidong Cheng contributed equally to this manuscript.

# Corresponding Author: (dxhlvqiang@163.com) (The First Affiliated Hospital of Nanjing Medical University)

**Affiliations:** Department of Urology, The First Affiliated Hospital of Nanjing Medical University.

Postal address: 300 Guangzhou Road, Nanjing 210029, P.R. China. Postal code: 210029. Tel: 86-25-83780079

**Supplementary Table.** Association between polymorphisms and OS of the patients

|  | Epirubicin | | | | MMC | | | |
| --- | --- | --- | --- | --- | --- | --- | --- | --- |
|  | Deaths | All patients | MST * | Log-rank *P* | Deaths | All patients | MST * | Log-rank *P* |
| *GSTP1* rs1695  AA | 1 | 78 | 38.4 | 0.886 | 2 | 61 | 33.5 | 0.878 |
| AG | 1 | 46 | 34.5 |  | 1 | 51 | 38.5 |  |
| GG | 0 | 6 | 37.0 |  | 0 | 2 | 88.5 |  |
| AA | 1 | 78 | 38.4 | 0.796 | 2 | 61 | 33.5 | 0.631 |
| AG+GG | 1 | 52 | 34.8 |  | 1 | 53 | 40.4 |  |
| *GSTO1* rs4925 |  |  |  |  |  |  |  |  |
| AA | 1 | 8 | 40.4 | 0.052 | 0 | 16 | 48.1 | 0.276 |
| AC | 0 | 40 | 33.6 |  | 2 | 30 | 33.0 |  |
| CC | 1 | 82 | 38.2 |  | 1 | 68 | 35.6 |  |
| AA | 1 | 8 | 40.4 | **0.018** | 0 | 16 | 48.1 | 0.485 |
| AC+CC | 1 | 122 | 36.7 |  | 3 | 98 | 34.8 |  |
| *GSTO2* rs156697 |  |  |  |  |  |  |  |  |
| CC | 1 | 15 | 36.7 | 0.186 | 0 | 21 | 46.0 | 0.420 |
| CT | 0 | 50 | 34.1 |  | 2 | 38 | 30.2 |  |
| TT | 1 | 65 | 39.2 |  | 1 | 55 | 37.6 |  |
| CC | 1 | 15 | 36.7 | 0.087 | 0 | 21 | 46.0 | 0.401 |
| CT+TT | 1 | 115 | 37.0 |  | 3 | 93 | 34.6 |  |
| *ABCB1* rs3747802 |  |  |  |  |  |  |  |  |
| TT | 2 | 126 | 36.2 | 0.967 | 3 | 111 | 37.0 | 0.963 |
| TC | 0 | 3 | 71.7 |  | 0 | 2 | 35.0 |  |
| CC | 0 | 1 | 21.0 |  | 0 | 1 | 8.0 |  |
| *ABCB1* rs3213619  TT  TC  CC | 2  0  0 | 114  9  7 | 37.8  36.6  23.7 | 0.868 | 3  0  0 | 103  6  5 | 34.3  53.0  66.4 | 0.841 |

* Mean survival time was calculated since the deaths were less than 50%.
